# Increasing abdominal pressure with and without PEEP: effects on intra-peritoneal, intra-organ and intra-vascular pressures

**DOI:** 10.1186/1471-230X-10-70

**Published:** 2010-07-04

**Authors:** Stephan M Jakob, Rafael Knuesel, Jyrki J Tenhunen, Richard Pradl, Jukka Takala

**Affiliations:** 1 Department of Intensive Care Medicine, University Hospital (Inselspital) and University of Bern, Bern, Switzerland; 2 Department of Intensive Care Medicine, Kuopio University Hospital, Kuopio, Finland

## Abstract

**Background:**

Intra-organ and intra-vascular pressures can be used to estimate intra-abdominal pressure. The aim of this prospective, interventional study was to assess the effect of PEEP on the accuracy of pressure estimation at different measurement sites in a model of increased abdominal pressure.

**Methods:**

Catheters for pressure measurement were inserted into the stomach, urinary bladder, peritoneal cavity, pulmonary artery and inferior vena cava of 12 pigs. The pressures were recorded simultaneously at baseline, during 10 cm H_2_0 PEEP, external abdominal pressure (7 kg weight) plus PEEP, external abdominal pressure without PEEP, and again under baseline conditions.

**Results (mean ± SD):**

PEEP alone increased diastolic pulmonary artery and inferior vena cava pressure but had no effect on the other pressures. PEEP and external abdominal pressure increased intraperitoneal pressure from 6 ± 1 mm Hg to 9 ± 2 mm Hg, urinary bladder pressure from 6 ± 2 mm Hg to 11 ± 2 mm Hg (p = 0.012), intragastric pressure from 6 ± 2 mm Hg to 11 ± 2 mm Hg (all p ≤ 0.001), and inferior vena cava pressure from 11 ± 4 mm Hg to 15 ± 4 mm Hg (p = 0.01). Removing PEEP and maintaining extraabdominal pressure was associated with a decrease in pulmonary artery diastolic but not in any of the other pressures. There was a significant correlation among all pressures. Bias (-1 mm Hg) and limits of agreement (3 to -5 mm Hg) were similar for the comparisons of absolute intraperitoneal pressure with intra-gastric and urinary bladder pressure, but larger for the comparison between intraperitoneal and inferior vena cava pressure (-5, 0 to -11 mm Hg). Bias (0 to -1 mm Hg) and limits of agreement (3 to -4 mm Hg) for pressure changes were similar for all comparisons

**Conclusions:**

Our data suggest that pressure changes induced by external abdominal pressure were not modified by changing PEEP between 0 and 10 cm H_2_0.

## Background

Intraabdominal hypertension (IAH), a sustained increase in intraabdominal pressure (IAP) above 12 mmHg, and abdominal compartment syndrome (ACS), a sustained increase in IAP above 20 mmHg with new-onset organ failure [1,2], are highly prevalent in critically ill patients, especially in those with a high body mass index, massive fluid resuscitation, and renal and coagulation impairment [3]. In this context, significant cardiovascular [4], respiratory [5], renal [6], hepatosplanchnic [7-10] and neurologic dysfunction [11] with increased mortality have been described [12].

Despite increasing evidence regarding the relevance of IAH and ACS, many intensive care units never measure the intraabdominal pressure (IAP), and no consensus exists on optimal timing of measurement or when decompressive laparotomy should be performed [13,14]. The definitions and diagnosis of IAH and ACS depend greatly on the accuracy, reliability and reproducibility of the IAP measurement technique [15], yet clinical measures of elevated IAP such as measuring abdominal girth and assessing the tenseness of the abdomen have low sensitivity and accuracy [13,16], and the direct measurement of IAP is rather invasive, and- with the exception of intraoperative measurements during laparoscopic interventions- not feasible in the clinical setting in most cases [17].

Since the abdomen and its contents can be considered primarily fluid in character, and consequently noncompressive, the IAP can be measured in nearly every part of the abdomen [18]. Alternative indirect methods for estimating IAP, such as measuring urinary bladder, inferior vena cava, gastric, intrarectal, and intrauterine pressures, have been used over the years. However, although the intravesical route has evolved as the gold standard [13], the ideal method for measuring intraabdominal pressure has increasingly become a matter of debate [18]. Both Foley manometers and IAP monitors are reliable and reproducible methods of measuring IAP, with low coefficients of variation, especially with increasing IAP [19].

While ACS may decrease lung compliance, increasing PEEP may limit the cranial expansion of the abdominal cavity. Pressure changes on both sides of the diaphragm may lead to alterations in the relationship between lower and upper intraabdominal, and intrathoracic and intraabdominal intravascular pressures [20]. The aim of this study was to assess the relationship between the pressure changes in the urinary bladder, the inferior caval vein, the stomach, and the pulmonary artery with directly measured intraperitoneal pressure changes during a small but clinically significant increase in IAP with and without moderate positive end-expiratory pressure (PEEP). We hypothesized that increased IAP decreases during PEEP release, and that this effect is more pronounced in the upper abdomen and intra-vascularly.

## Methods

This study was approved by the Institutional Animal Care and Use Committee of the University of Kuopio, Finland.

### Anesthesia and animal preparation

Twelve female pigs weighing 34 (27-43) kg (median, range) were deprived of food but had free access to water 12 h before the experiment. After premedication with atropine (0.05 mg/kg) and azaperone (8 mg/kg intramuscularly), an ear vein was cannulated and thiopental sodium (5-15 mg/kg) was administered intravenously for endotracheal intubation. Anesthesia was maintained with thiopental (5 mg/kg/h) and fentanyl (30 mg/kg/h) until the end of the surgical procedure, and afterwards with thiopental (5 mg/kg/h) and fentanyl (5 mg/kg/h) until the end of the experiment.

The animals were ventilated with a volume-controlled ventilator (Servo 900C, Siemens AG, Solna, Sweden) without positive end-expiratory pressure. Fractional inspired O_2 _concentration was adjusted to reach a target arterial PO_2 _of 100 mmHg. Tidal volume was kept at 10 ml/kg, and the minute ventilation was adjusted to maintain arterial PCO_2 _levels between 33 and 45 mmHg.

A pulmonary artery catheter (CO Catheter, Edwards Lifesciences, Irvine, CA, USA) was inserted using pressure readings via the right submandibular vein. A femoral artery catheter and a gastric air tonometer with connections to pressure transducers were inserted (Tonometrics, Worcester, MA). Another catheter was inserted into the inferior vena cava via the right internal jugular vein, and its correct position (tip at lower border of liver) was confirmed by ultrasound. The abdominal wall was punctured with an 18 G needle in the right lower abdominal quadrant. Injuries to the gut and intraabdominal organs were avoided by lifting the abdominal wall with two towel clamps. A commercially available 16 G × 30 cm single-lumen intravenous catheter (Arrow International, Reading, PA, USA) was inserted into the peritoneal cavity using a J-shaped guide wire and a dilator. A Foley catheter was placed in the urinary bladder via the urethra. These catheters were also connected to pressure transducers.

During surgery the animals received saline at 5 ml/kg/h. Additional Ringer's lactate solution and hydroxyethyl starch were administered in equal amounts during surgery to keep the pulmonary artery occlusion pressure between 6 and 12 mmHg. The body temperature of the animals was kept at 38 ± 1°C using an operating table heater and warmed fluids. The position of the catheters was checked by palpation and by direct visualization after the experiments had been completed.

### Hemodynamic monitoring

**Figure 1:**
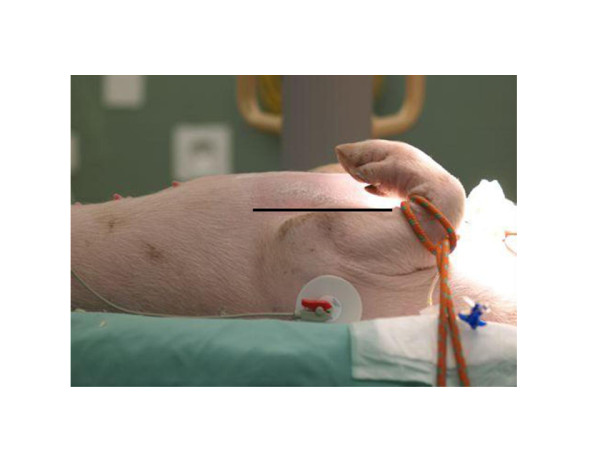
**Zero reference for pressure transducers**. Black line: zero level.

All pressures were measured continuously with quartz pressure transducers and displayed on a multimodular monitor and recorder (AS3, Datex-Ohmeda, Helsinki, Finland). At the same time, the signals were recorded with a computer program (Windaq 1.60; Dataq Instruments Inc., Akron, OH, USA) for later analysis. All pressure transducers were simultaneously zeroed to a level corresponding to the ventral border of the front leg (Figure 1). Heart rate was measured from the electrocardiogram, which was also continuously monitored. Cardiac output was measured with the thermodilution technique (mean value of three measurements). Central venous blood temperature (°C) was recorded from the thermistor of the pulmonary artery catheter.

### Experimental protocol

After placement of the catheters and hemodynamic stabilization for 30 minutes, the urinary bladder was emptied and refilled with 50 ml of normal saline. Afterwards, baseline values were taken. Next, abdominal and end-expiratory pressures were changed in four non-randomized steps, each lasting approximately 10 minutes: 1) increase in PEEP to 10 cm H_2_O; 2) increase in abdominal pressure by applying an external abdominal weight of 7 kg; 3) decrease in PEEP to 0 cm H_2_O; 4) removal of the abdominal weight. Each change in pressure was followed by approximately five minutes of stabilization before the measurements were taken. Artefact-free recordings of two minutes were averaged. At the end of this experiment, the animals were subsequently included in a separate study on hepato-splanchnic blood flow regulation [21].

### Statistics

Statistical analysis was performed with the SPSS software (version 12.01, SPSS Inc., Chicago, IL, USA). Parametric tests were used. Pressures at baseline were compared using one-way ANOVA. ANOVA for repeated measurements was applied for the assessment of pressure changes at the different locations. Effects of PEEP, and of external abdominal pressure with and without PEEP, were compared to baseline post-hoc using paired T tests. The same test was used to assess differences between external abdominal pressure with and without PEEP. The Bonferroni approach was applied to compensate for multiple (n = 4) comparisons: for T tests, a p value of 0.0125 was considered significant. To compare the magnitude of pressure changes at the different locations, one-way ANOVA and linear correlation was used. Data are presented by means of Bland-Altman and correlation plots. Bland-Altman statistics were calculated using Microsoft Office Excel 2007 (Microsoft Corporation, Wallisellen, Switzerland).

## Results

In one animal the intraperitoneal pressure did not increase after applying external weight onto the abdomen; consequently this animal was excluded from statistical analysis but an additional experiment was performed. Due to technical reasons or evidence of incorrect placement of the catheters after the experiment, some pressures are missing (inferior vena cava: n = 2; stomach: n = 2; pulmonary artery: n = 3; systemic artery: n = 2).

### Systemic hemodynamics

Systemic hemodynamics remained stable during the experiment (Table 1).

**Table 1:** Systemic hemodynamics and central temperature.

	Baseline*	PEEP	PEEP+external abdominal pressure*	External abdominal pressure*	End of experiment	P*	*P*^*#*^
**Heart rate (beats/min)**	80 ± 14	80 ± 19	81 ± 23	85 ± 28	83 ± 17	***0.916***	

**Cardiac output (ml/kg/min)**	101 ± 24				96 ± 20		***0.371***

**Systemic mean arterial pressure (mm Hg)**	87 ± 16	84 ± 16	91 ± 18	79 ± 23	84 ± 16	***0.164***	

**Central venous pressure (mmHg)**	8 ± 3				8 ± 3		***0.808***

**Pulmonary capillary occlusion pressure (mmHg)**	8 ± 3				7 ± 2		*0.615*

**Central temperature (°C)**	37.3 ± 1.2				37.5 ± 1.3		*0.337*

Values are mean ± SD.

*ANOVA for repeated measurements. ^#^Paired T Test.

### Absolute pressures at baseline

At baseline, there were significant differences between pressures at the different measurement sites (p < 0.001; Table 2).

**Table 2:** Intraperitoneal, intraorgan and intravascular pressures (mm Hg) during the experiment.

	Baseline	PEEP	PEEP+external abdominal pressure	External abdominal pressure	End of experiment	P*
**Intraperitoneal pressure**	6 ± 1	6 ± 1	9 ± 2^##^	9 ± 2^##^	5 ± 1	***<0.001***

**Urinary bladder pressure**	6 ± 2	7 ± 2	11 ± 2^##^	10 ± 2^##^	6 ± 1	***<0.001***

**Intragastric pressure**	6 ± 2	8 ± 2	11 ± 2^##^	10 ± 2^##^	6 ± 2	***<0.001***

**Inferior vena cava pressure**	11 ± 4	12 ± 3^#^	15 ± 4^#^	14 ± 3^#^	11 ± 3	***<0.001***

**Diastolic pulmonary artery pressure**	14 ± 5	16 ± 4^#^	17 ± 5	16 ± 5^&^	14 ± 6	***0.003***

Values are mean ± SD.

*: ANOVA For Repeated Measurements. Paired T Test, vs. Baseline, ^#^P ≤ 0.01, ^##^P ≤ 0.001; External Abdominal Pressure +PEEP vs. -PEEP, ^&^p = 0.007.

### Effects of PEEP and external abdominal pressure on intra-abdominal pressures

PEEP had no effect on either intraperitoneal, intragastric or urinary bladder pressure (Table 2). All of these pressures increased with extraabdominal pressure, independent of the presence or absence of PEEP (Table 2).

### Effects of PEEP and external abdominal pressure on intra-vascular pressures

PEEP was associated with an increase in both intravascular pressures (Table 2). Inferior vena cava but not pulmonary artery pressure increased during external abdominal pressure (Table 2).

### Comparison between absolute pressures and pressure changes

**Figure 2:**
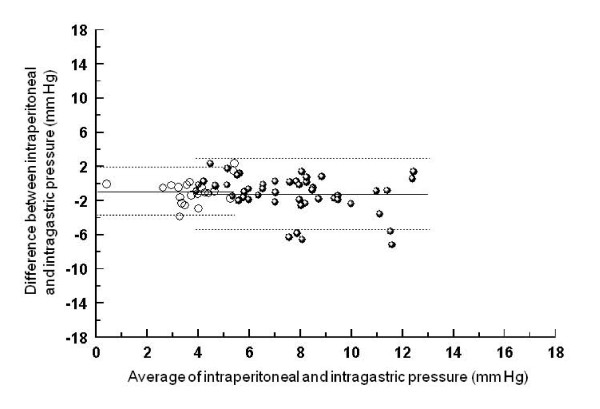
**Bland-Altman graph for the comparison between intraperitoneal and intragastric pressure**. Absolute values are displayed on the right side of the figure, and changes between baseline and external abdominal pressure with and without PEEP on the left side. Full lines represent bias (average of the differences), and dotted lines limits of agreement (+/- 1.96 SD).

**Figure 3:**
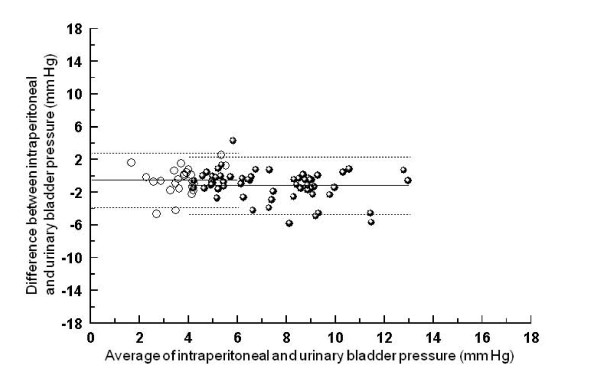
**Bland-Altman graph for the comparison between intraperitoneal and urinary bladder pressure**. Absolute values are displayed on the right side of the figure, and changes between baseline and external abdominal pressure with and without PEEP on the left side. Full lines represent bias (average of the differences), and dotted lines limits of agreement (+/- 1.96 SD).

**Figure 4:**
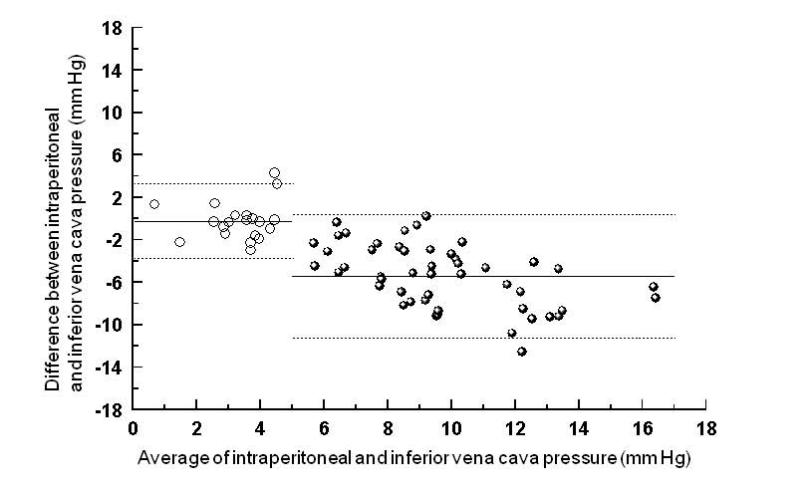
**Bland-Altman graph for the comparison between intraperitoneal and inferior vena cava pressure**. Absolute values are displayed on the right side of the figure, and changes between baseline and external abdominal pressure with and without PEEP on the left side. Full lines represent bias (average of the differences), and dotted lines limits of agreement (+/- 1.96 SD).

Pressures and changes in pressures are compared in Figures 2, 3 and 4. Bias and limits of agreement were similar for the comparisons of absolute intraperitoneal pressure with intra-gastric (-1, 3 to -5 mm Hg) and urinary bladder pressure (-1, 2 to -5 mm Hg), but larger for the comparison between intra-peritoneal and inferior vena cava pressure (-5, 0 to -11 mm Hg). Bias (0 to -1 mm Hg) and limits of agreement (3 to -4 mm Hg) for pressure *changes *were similar for all three comparisons (Figures 2, 3, 4, lefthand side).

**Figure 5:**
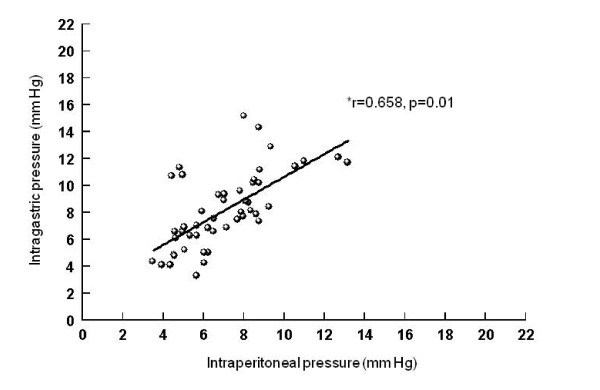
**Correlation between intraperitoneal and intragastric pressure**. *Pearson correlation.

**Figure 6:**
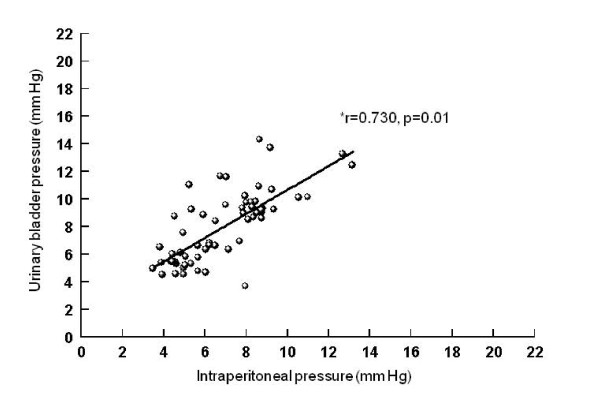
**Correlation between intraperitoneal and urinary bladder pressure**. *Pearson correlation.

**Figure 7:**
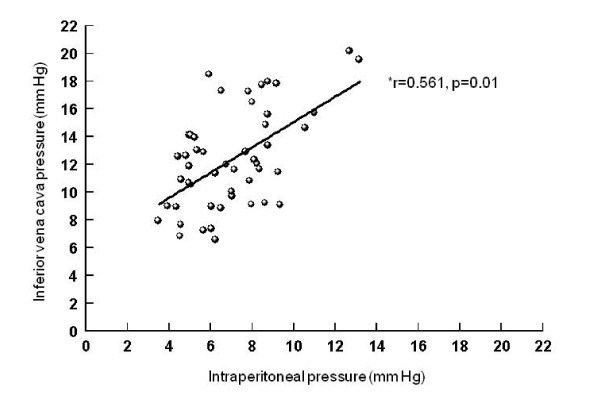
**Correlation between intraperitoneal and inferior vena cava pressure**. *Pearson correlation.

**Figure 8:**
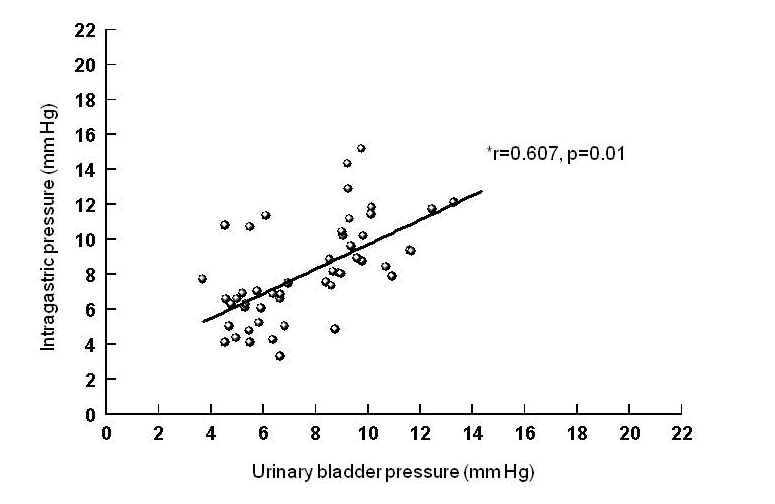
**Correlation between urinary bladder and intragastric pressure**. *Pearson correlation.

**Figure 9:**
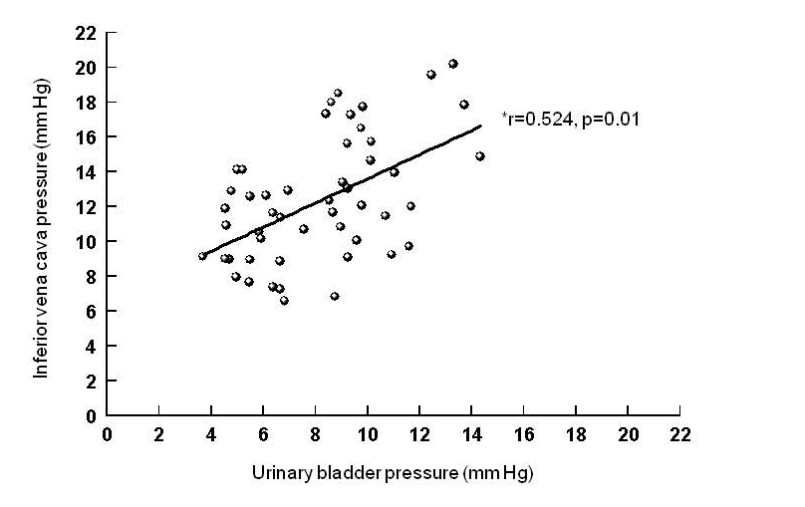
**Correlation between urinary bladder and inferior vena cava pressure**. *Pearson correlation.

**Figure 10:**
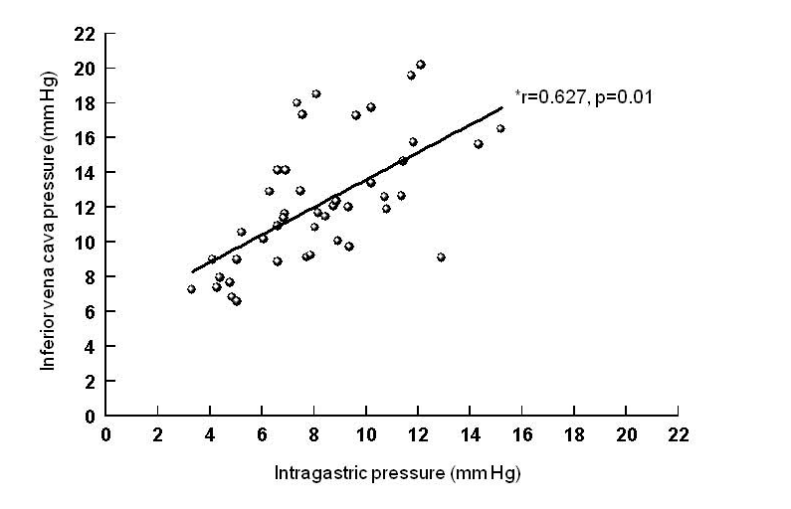
**Correlation between intragastric and inferior vena cava pressure**. *Pearson correlation.

**Figure 11:**
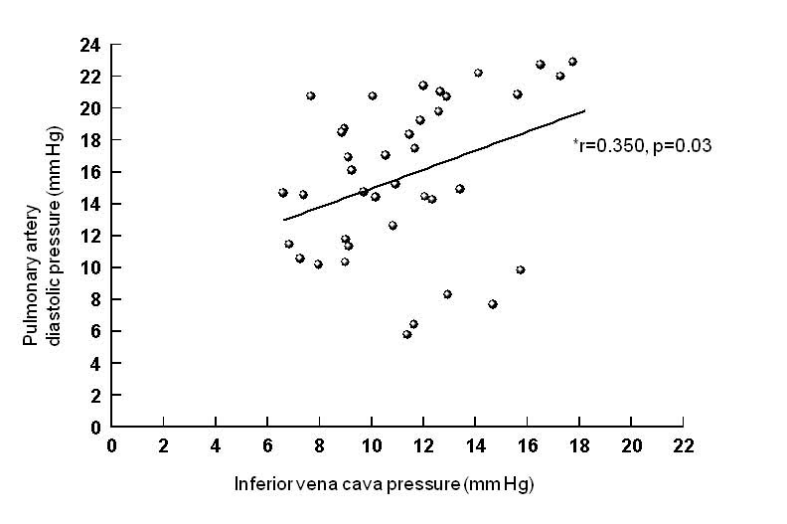
**Correlation between inferior vena cava and pulmonary artery diastolic pressure**. *Pearson correlation.

Correlations between intraperitoneal and intragastric, urinary bladder and inferior vena cava pressures (Figures 5, 6, 7), between urinary bladder and intragastric and inferior vena cava pressures (Figures 8 and 9), between intragastric and inferior vena cava pressures (Figure 10), and between inferior vena cava and pulmonary artery diastolic pressures (Figure 11) were all significant, although at varying degrees. Intraperitoneal and urinary bladder pressures correlated best (r = 0.730, p = 0.01).

## Discussion

The main finding of this study is that changing PEEP between 0 and 10 cm H_2_0 did not modify the pressure changes induced by external abdominal pressure intraperitoneally, in urinary bladder and stomach, and in inferior vena cava.

Normal IAP is 5-7 mm Hg in non-morbidly obese patients [22]. During spontaneous respiration, inspiration and expiration are controlled by opposing activity of the diaphragm and abdominal muscles, which varies the shape of the pressurized abdominal cavity [23]. Accordingly, IAP does not increase. Situations in which IAP increases and pressure measurement has been advocated are non-operative management of blunt hepatic trauma [24], abdominal surgery [25], and abdominoplasty [26]. IAH at ICU admission is associated with severe organ dysfunction during the intensive care unit stay [27]. IAP, and especially abdominal perfusion pressure (mean arterial pressure - IAP), appears to be a clinically useful resuscitation endpoint and predictor of patient survival during treatment for IAH and ACS [2].

In the present study, median IAP values increased by approximately 5 mm Hg and just reached the lower limit for the diagnosis of IAH [1]. In agreement with others, we found similar increases and decreases in intra-abdominal, intra-vesical and intra-gastric pressures with changing abdominal pressure [28-31].

In this model without lung injury, 10 cm H_2_0 PEEP had no significant effect on the relationship between the different pressures. In patients with acute lung injury, an increase and decrease in PEEP from 8-13 H_2_O and back did not change total hepato-splanchnic blood flow or the gastric mucosal-arterial pCO_2 _gradient [32]. In contrast, in patients without acute lung injury, perioperative application of PEEP reduced splanchnic blood flow [33,34]. Application of PEEP has also been associated with marked reduction of total hepatic and portal venous blood flow in various experimental models, although the results are controversial [35-38]. It is possible that higher PEEP levels in our study would have increased IAP.

The present study was not designed to assess effects of intra-thoracic and IAP changes on perfusion. In pigs and dogs, the effect of low IAP (7-8 mm Hg) on splanchnic perfusion is minimal, while at higher IAP values (14-16 mm Hg) portal blood flow decreases [39,40].

Increases in IAP are also associated with increased respiratory pressures, and decreased lung compliance and gas exchange [41,42]. In our study, the increase in intra-thoracic pressure with increased abdominal pressure, if any, did not translate into a significant increase in pulmonary artery diastolic pressure. However, inferior vena cava pressure was higher than intraabdominal and intraorgan pressures. Some [40] but not all [29,43] researchers found higher inferior vena cava pressures than IAP. A somewhat increased intraabdominal intravenous pressure seems physiological since a pressure gradient from inside to outside the vessel prevents vessel collapse, and a driving pressure is needed to generate flow to the heart. Interestingly, both the increase in IAP and application of PEEP resulted in an increase in intravascular pressure in the inferior vena cava. While the increase in intravascular pressure is the result of increased pressure around the vein in the former situation, it is a consequence of intravascular pressure transmission in the latter. This is evident from the unchanged IAP when only PEEP was applied. In humans with increased IAP, intravascular pressure in the inferior vena cava was not reflected by a similar increase in the superior caval vein pressure, due to a "waterfall" effect [44,45]. The increase in IAP evoked a transition of the abdominal venous compartment from a zone 3 to a zone 2 condition, presumably impairing venous return despite an increased pressure gradient between the abdominal and thoracic compartments [44]. This can be explained by decreased femoral vein blood flow [29]. Accordingly, PEEP *and *increased IAP seems to be the most crucial of the studied circumstances for venous return [46].

It has been shown that bolus administration of opioids and muscle relaxants can increase and decrease, respectively, IAP [47,48]. Furthermore, body position also has an effect on IAP [49]. Since we did not administer drugs as boli and kept the animals in supine position throughout the experiments, we can exclude such effects on our measurements. Nevertheless, when results of different studies are compared, these aspects need to be considered.

A limitation of the experimental set-up is the use of external abdominal pressure, and the fact that it produced only moderate increases in the measured pressures. In humans, external abdominal pressure has been used to increase intraabdominal pressure [50]. There is considerable variation in the literature regarding the design for ACS in animals [51], and recommendations for research regarding IAH and ACS have been published only recently [52]. Consequently, our findings cannot necessarily be extrapolated to clinical situations with IAH. Nevertheless, it has been shown that increases in intra-gastric pressure or IAP of only a few mm Hg, e.g., during prone positioning [53,54] or closure of laparotomy [55], can worsen gastric-mucosal perfusion, diuresis and arterial pO_2_.

Even if the changes in pressures were similar at the different locations, the absolute values differed. Such pressure differences may be real or the result of technical circumstances. As an example, instillation of 50 ml into the pig's bladder may be too much, as in humans a maximal instillation volume of 20 ml has been recommended. Gudmundsson et al. [29] demonstrated that the volume of fluid needed to increase porcine intra-vesical pressure by 2 mm Hg varies widely and is dependent on IAP. On the other hand, Fusco et al. [56] showed that instillation of 50 ml of fluid into the bladder improves the accuracy of the intra-vesicular pressure in measuring elevated IAP.

A further limitation is that we measured neither esophageal pressure nor the impact of tidal ventilation on changes in any of the measured pressures. Recent data suggest that the compliance of the abdominal wall has an impact on the magnitude of IAP changes during tidal ventilation [57].

## Conclusions

Our data suggest that pressure changes induced by external abdominal pressure were not modified by changing PEEP between 0 and 10 cm H_2_0. Inferior vena cava pressure overestimated intra-peritoneal pressure.

## Competing interests

The Department of Intensive Care Medicine has, or has had in the past, research contracts with Abbott Nutrition International, B. Braun Medical AG, CSEM SA, Edwards Lifesciences Services GmbH, Kenta Biotech Ltd, Maquet Critical Care AB, Omnicare Clinical Research AG, and Orion Corporation; and research & development/consulting contracts with Edwards Lifesciences SA and Maquet Critical Care AB. The money is/was paid into a departmental fund; no author receives/received individual fees. The past contract with Edwards Lifesciences is unrelated to and did not influence the current study.

## Authors' contributions

SMJ and JT designed the study. SMJ, JJT and RP performed anesthesia, surgery, and carried out the experimental protocol. RK analyzed the data and drafted the manuscript. SJ performed the statistical analysis. SMJ and JT critically revised the manuscript. All authors read and approved the final manuscript.

## Pre-publication history

The pre-publication history for this paper can be accessed here:

http://www.biomedcentral.com/1471-230X/10/70/prepub
